# Combining dendritic cells and B cells for presentation of oxidised tumour antigens to CD8^+^ T cells

**DOI:** 10.1038/cti.2017.28

**Published:** 2017-07-07

**Authors:** Melanie L Grant, Nicholas Shields, Silke Neumann, Katrin Kramer, Andrea Bonato, Christopher Jackson, Margaret A Baird, Sarah L Young

**Affiliations:** 1 Pathology Department, Dunedin School of Medicine, University of Otago, Dunedin, New Zealand; 2 Ambulatorio Veterinario Summano, Via Europa, Santorso, Italy; 3 Department of Medicine, Dunedin Hospital, Dunedin, New Zealand

## Abstract

The dendritic cell (DC) is the foremost antigen-presenting cell (APC) for *ex vivo* expansion of tumour-specific patient T cells. Despite marked responses in some patients following reinfusion of DC-activated autologous or HLA-matched donor T cells, overall response rates remain modest in solid tumours. Furthermore, most studies aim to generate immune responses against defined tumour-associated antigens (TAA), however, meta-analysis reveals that those approaches have less clinical success than those using whole tumour cells or their components. Tumour lysate (TL) is used as a source of tumour antigen in clinical trials and potentially represents the full range of TAAs in an undefined state. Little is known about how different APCs cooperate to present TL antigens. We examined the effect of oxidised whole-cell lysate (ox-L) versus soluble fraction freeze–thaw lysate (s-L) on bone marrow-derived DCs and macrophages, and magnetic bead-isolated splenic B cells. The APCs were used individually, or in combination, to prime T cells. CD8^+^ T cells produced interferon (IFN)-γ in response to both s-L and ox-L, but only proliferated in response to ox-L. IFN-γ production and proliferation was enhanced by priming with the DC+B cell combination. Compared to DC alone, a trend toward greater interleukin (IL)-12 production was observed when DC+B cell were loaded with s-L and ox-L antigens. CD8^+^ T-cell specific lysis *in vivo* was greatest in ox-L-primed groups and DC+B cell priming significantly increased *in vivo* cytotoxicity compared to DC alone. These improved T-cell responses with two APCs and stressed cell lysate has implications for APC-based adoptive cell therapies.

A cancer treatment tailor-made and specific to each cancer patient regardless of haplotype, genotype or immunodominant peptide(s) is the Holy Grail of cancer immunotherapy.^1^ Lysate generated from the patient’s tumour has the potential to meet these conditions. Tumour lysate provides a source of all potential tumour antigens: immuno-dominant antigens, known cancer-specific antigens, patient-specific neo-antigens and antigens that are as yet unidentified. Tumour lysate contains CD4 and CD8 epitopes that can stimulate both arms of the T-cell-mediated response. The major drawback with autologous lysate is that it also comprises self-antigens, which can trigger immunosuppressive tolerance mechanisms. In order to generate a strong anti-tumour response against tumour lysate antigens, tolerance may need to be overcome. This carries a concurrent risk of auto-immune side-effects, however, to date the risk of autoimmunity induction with the use of lysate appears to be small,^2, 3, 4, 5, 6, 7, 8^ and, in the case of melanoma at least, appears necessary for successful tumour control.^9, 10^

Breaking open cells by freeze–thaw lysis exposes normally hidden intracellular molecules such as HMGB1, calreticulin,^11, 12, 13^ ATP, uric acid, nucleic acids and lipids. APCs respond to these compounds via toll like receptors (TLRs), activating ‘danger’ and stress signal pathways.^14^ Freeze–thaw lysis is commonly used to generate a necrotic-type cell death of tumour cells in the clinic; however this lysate can be immunosuppressive. *In vivo* lysis of cancer cells does occur, but at levels that may be insufficient to attract the attention of the immune system. The larger quantities of lysed cells in tumour lysate may provide a more potent source of danger and stress signals for APC activation. Furthermore, recent studies comparing different methods of lysate generation have shown that hypochlorous acid (HOCl)-oxidation of cells prior to freeze thaw lysis improved the immunogenicity of oxidised tumour lysate in ovarian cancer patients, and that this method of lysate pre-treatment was superior to heat or acid stress.^2, 15, 16^

The melanoma cell line B16.OVA was chosen for the experiments in this study, as it is a poorly immunogenic and highly aggressive tumour when employed in *in vivo* experiments. These features make it a difficult target in immunotherapy, reflecting the generally poorly immunogenic and aggressive tumours in patients that are refractory to treatment. This study compared oxidised B16.OVA tumour lysate with the soluble fraction of B16.OVA lysate as antigen sources for APC presentation.

In this study, we utilised GM-CSF-differentiated, bone-marrow-derived DCs (GMDC) as an approximation of monocyte-derived DCs (mo-DCs) currently used in the clinic. Many clinical trials have demonstrated the safety and efficacy of GM-CSF-differentiated mo-DC-based immunotherapies, but robust responses have been limited.^3, 17, 18, 19, 20, 21, 22, 23, 24^ While many studies are focused on improving monocyte-derived DC effectiveness and exploring the utility of different populations of DCs, other studies are noticing the T-cell priming capabilities of other immune cells. Macrophages (Mϕ)^25, 26, 27, 28^ B cells,^29, 30, 31, 32, 33, 34^ neutrophils^35, 36, 37, 38, 39^ and even eosinophils^40^ are all being assigned various primary and accessory roles in antigen presentation and the systems biology nature of antigen presentation and T cell activation is beginning to be delineated. We have examined whether GMDC-mediated ACT might be improved by combining these DCs with other professional APCs.

We loaded GMDCs, Mϕs and B cells, alone and in combination, with the soluble fraction of freeze–thaw lysate (soluble lysate/s-L) and with hypochlorous acid-oxidised whole freeze–thaw lysate (oxidised lysate/ox-L). We compared the surface markers associated with antigen presentation and co-stimulation on the APCs, as well as their interleukin (IL)-12 production. The costimulatory molecule CD40 was upregulated on GMDCs in response to oxidised lysate, while CD86 but not CD40 was upregulated on B cells, demonstrating differential responses by the APCs to the lysate components. The combination of a GM-CSF-generated APC (DC) and a B-cell yielded up to three times the amount of IL-12 compared to lysate-pulsed DCs alone.

We also assessed the APCs’ capacity for activating T-cell proliferation, interferon (IFN)-γ production and *in vivo* cytotoxicity. Our data demonstrated that IFN-γ and IL-12 were produced in response to both soluble and oxidised B16.OVA melanoma cell lysates. However, CD8^+^ T cells only proliferated *in vitro* in response to oxidised lysate and *in vivo* cytotoxicity was likewise greater in response to oxidised lysate. Moreover, CD8^+^ T-cell *in vitro* proliferation and *in vivo* cytotoxicity was enhanced when T cells were primed by the DC+B cell combination.

These results have implications for adoptive cell therapy, which may be enhanced by 1. Not relying exclusively on GMDC/mo-DCs for *ex vivo* priming of patient T cells and 2. Stressing tumour cells by oxidation prior to loading onto APCs. Given that patient DCs constitute a rare population, which cannot be expanded *ex vivo*, the inclusion of properly activated B cells when priming patient T cells may make generating sufficient CTL numbers for ACT easier as well as rendering them more effective. This issue is particularly pertinent in pediatric cell-mediated immunotherapy where small blood volumes make sufficient DC numbers even more challenging.

## Results

### GMDC, Mϕs and B cells vary in their surface phenotype response to soluble and oxidised lysates

**Figure 1:**
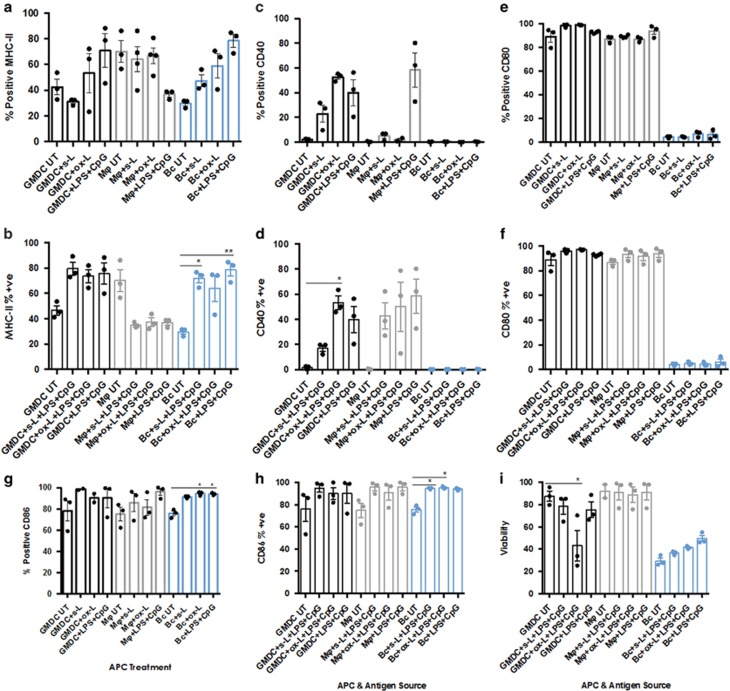
Activated GMDC, Mϕ and B cells vary in their activation and viability response to soluble and oxidised lysates. Bone marrow-derived precursor cells were incubated for 10 days in GM-CSF (5 ngml^−1^)+IL-3 (5 ngml^−1^)+FCS10% (Mϕ) or 6 days in GM-CSF (20 ng ml^−1^)+FCS (5%) (GMDC). B cells were isolated by anti-CD43 magnetic bead isolation as described in Materials and Methods section. APCs were pulsed overnight with s-L and ox-L (1:1 ratio tumour cell:APC)+LPS&CpG. After 24 h cells were collected, stained with live/dead exclusion dye (Live Dead Near Infrared, Life Technologies) and labelled with surface mABs against MHC-II, CD40, CD80 and CD86. Cells were fixed with 4% paraformaldehyde, stored overnight at 4 °C and collected by Flow Cytometry the next day (Gallios, Beckman Coulter). Data were analysed on FlowJo Version X (TreeStar) and graphed in Prism (GraphPad). (**a**–**h**) Summary data of three independent experiments showing MHC-II and costimulatory molecule expression on GMDC, Mϕ and B cells in response to s-L and ox-L. (**i**) Summary data of GMDC, Mϕ and B-cell viability after 24-h exposure to LPS&CpG or s-L and ox-L+LPS&CpG. Statistically significant differences calculated using Kruskal–Wallis followed by Dunn’s test for multiple comparisons with no Bonferroni correction *P<0.05. Error bars=mean±s.e.m.

Unactivated B cells are known to be poor APCs whereas B-cell activation triggers antigen uptake and enhanced presentation. Proper activation of DCs and Mϕs is also vital for ensuring optimal T-cell activation. We therefore compared DCs, Mϕs and B cells for their ability to upregulate MHC Class II and the costimulatory molecules CD40, CD80 and CD86 (Figures 1a–d) in response to the two lysates±LPS&CpG. (Supplementary Figure 2 shows the gating strategy used to phenotype DCs and Mϕs. See Supplementary Figure 3 for B-cell isolation and phenotyping). MHC-II was not upregulated on GMDCs, Mϕs or B cells in response to either lysate alone (data not shown), but only in the presence of LPS&CpG. However, no statistically significant changes in MHC-II per cent positivity were observed in GMDC or Mϕs in response to lysate+LPS&CpG (Figure 1a). There was a statistically significant upregulation of MHC-II expression by B cells in response to soluble lysate+LPS&CpG, however this increase was not greater than that observed in response to LPS&CpG, indicating that the increase was due to the action of LPS&CpG and not the lysate antigens.

CD40 expression on GMDC was likewise significantly upregulated in response to ox-L in the absence of LPS&CpG (*P*<0.05; Figure 1b). When loaded with ox-L+LPS&CpG there was no additional increase in CD40 expression (Figure 1c). In Mϕs, no upregulation of CD40 was observed in the presence of either lysate alone (data not shown). The Mϕ+lysate+LPS&CpG data varied widely and the increased CD40 was also due to the effect of LPS&CpG (Figure 1c). For B cells no increase in CD40 was observed in the presence of lysate ±LPS&CpG (Figure 1c).

The per cent positivity of CD80 was constitutively high on DCs and Mϕs and no changes were observed in response to lysates alone (data not shown) or lysates+LPS&CpG (Figure 1d). CD80 expression was low on untreated B cells and this did not change in response to lysates or LPS&CpG.

Finally, the expression of CD86 was high on the three untreated APCs and no statistically significant increases in CD86 were observed in response to soluble or oxidised lysate±LPS&CpG on GMDC or Mϕ (Figure 1f). However, the increase in expression on B cells was significant (*P*<0.05). Notably, these increases were observed in the presence of s-L and ox-L only (Figure 1e) and therefore were not attributable to the actions of LPS&CpG. indicating that components in the lysate were able to activate the B cells.

These data demonstrated differences in these APCs’ responses to the lysates in terms of their ability to stimulate antigen presentation and co-stimulation capacities. Mϕ displayed reduced MHC-II and no increase in costimulatory capacity. LPS&CpG-activated GMDC and B cells, by contrast, both displayed increased antigen presentation capacity, with material in the ox-L stimulating B-cell MHC-II upregulation. Oxidised lysate likewise contained properties capable of stimulating CD40 upregulation on GMDC, even in the absence of LPS&CpG. On B cells CD86 expression was upregulated in the presence of both soluble and oxidised lysates ±LPS&CpG (*P*<0.05), again demonstrating that oxidised lysate alone could stimulate this B-cell response. Thus GMDC and B cells demonstrated superior capacities for driving a T-cell response to tumour lysate antigens, particularly oxidised lysate antigens.

### Variation in APC viability in response to lysates

It has been noted previously that tumour lysate was not toxic to DCs,^41^ however, we noted differences in the ability of soluble and oxidised lysates to induce APC death (Figure 1g). GMDC loaded with oxidised lysate+LPS+CpG displayed significant cell death (*P*<0.05). As expected, untreated B-cell viability was greatly reduced after overnight culture and the slight increase in viability observed in the presence of lysate or LPS&CpG was not statistically significant. Mϕs, by contrast, retained excellent viability after exposure to both lysates, irrespective of whether they had been activated with LPS+CpG.

### Oxidised lysate stimulates more CD8^+^ T-cell proliferation than soluble lysate when presented by GMDC+B cells

**Figure 2:**
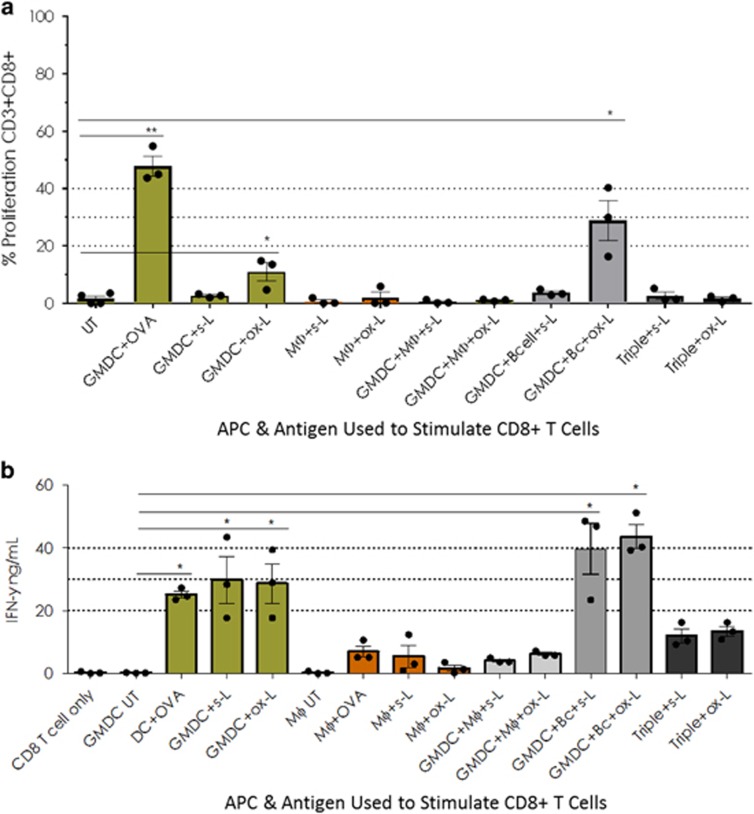
Oxidised lysate stimulates greater CD8^+^ T-cell proliferation than soluble lysate when presented by GMDC+B cell, but GMDC+B cell induces no increase in CD8^+^ T-cell IFN-γ production over GMDC alone when presenting lysate antigens. Day 6 GMDC, Day 10 Mϕ and freshly isolated splenic B cells (CD43− cells), or combinations thereof, were pulsed overnight with whole OVA protein (50 μg ml^−1^) and B16.OVA s-L or ox-L (1:1 ratio, tumour cell:APC). LPS (1 μg ml^−1^) and CpG (0.3 μg ml^−1^) were added at the same time as the lysates. The following morning CFSE-labelled CD8^+^ T cells were added (1:10 ratio, APC:T cell). APCs and T cells were co-cultured for 72 h, conditioned cell media collected prior to cell harvest and stored at −20 °C. IFN-γ levels were assessed by anti-IFN-γ ELISA. Data were analysed in Excel and graphed in Prism (GraphPad). Cells were stained with dead cell exclusion dye labelled with surface antibodies against CD11c, CD19 (dump channel), CD3 and CD8. Cells were fixed in 4% paraformaldehyde, stored overnight at 4 °C and acquired the following day on a Gallios Flow Cytometer (Beckman Coulter). Data were analysed on FlowJo version X (TreeStar) and graphed in Prism (GraphPad). (**a**) Summary data of three independent experiments showing CD8^+^ T cell proliferation at 72 h. (**b**) Summary data of three independent experiments showing IFN-γ levels in CD8^+^ T cell cultures at 72 h. Statistically significant differences calculated using Kruskal–Wallis followed by Dunn’s test for multiple comparisons with no Bonferroni correction *P<0.05. Error bars=mean±s.e.m.

Having assessed the APC response to the lysates, we next assayed CD4^+^ and CD8^+^ T-cell proliferation and IFN-γ production after presentation of soluble and oxidised lysate antigens by these APCs and combinations thereof. No improvement in CD4^+^ T cell proliferation was observed in any of the APC combination groups when presenting soluble or oxidised lysate antigens (data not shown). When unactivated, lysate-loaded APCs presented soluble lysate antigens to CD8^+^ T cells any combination of APCs yielded an improved proliferation response over presentation by GMDC alone (data not shown). No combination was superior to any other in these experiments; however the B cell+Mϕ combination was eliminated as not useful. By contrast when oxidised lysate was used as the antigen source the GMDC+B cell group elicited the greatest T-cell proliferation, although this difference did not reach statistical significance (Figure 2a).

### The combination of GMDC+B-cell induces no increase in CD8^+^ T-cell IFN-γ production over GMDC alone when presenting lysate antigens

Since T-cell proliferation does not necessarily correlate with function, lysate-loaded GMDCs, Mϕs, B cells and combinations thereof, were compared for their ability to stimulate production of the key inflammatory cytokines IFN-γ, TNF-α and the regulatory cytokine IL-10 in APC- CD4^+^ and APC- CD8^+^ T-cell co-cultures. Soluble TNF-α was not detected in any groups (data not shown). IL-10 was very low (<1 ng ml^−1^) in all cultures, with levels in oxidised lysate-primed CD8^+^ T-cell cultures consistently lower (0.7 ng ml^−1^ or less) than their counterpart soluble lysate-primed cultures (data not shown).

In CD4^+^ T-cell cultures no APC combination yielded any improvement in IFN-γ production over GMDC alone (Supplementary Figure 1). In CD8^+^ T-cell co-cultures the GMDC+B-cell groups yielded the greatest IFN-γ in response to both soluble and oxidised lysate, however, the increase over GMDC-stimulated CD8^+^ T cells did not reach statistical significance (*P*<0.06 when analysed by Kruskal–Wallis followed by Dunn’s test without Bonferroni correction for multiple comparisons; *P*>0.99 with Bonferroni correction; Figure 2b).

### GMDC+B cell-primed CD8^+^ T cells induce superior *in vivo* cytotoxicity over GMDC alone in response to both soluble and oxidised lysate antigens

**Figure 3:**
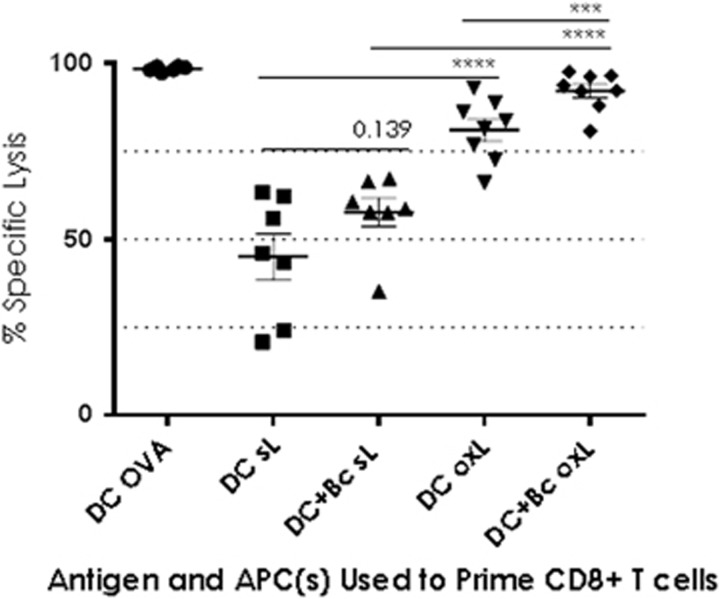
GMDC+B cell-primed CD8^+^ T cells induce superior *in vivo* cytotoxicity over GMDC alone in response to oxidised lysate antigens. Day 6 GMDC, ±spleen-derived B cells, were pulsed overnight with whole OVA protein (50 μg ml^−1^) and s-L or ox-L (1:1 ratio, tumour cell:APC). The following day CD4^+^ and CD8^+^ T cells were added to the APCs (10:1) and co-cultured for 3 or 4 days. Lysate-primed effector T cells were injected i.v. into C57BL/6 mice. The following day C57BL/7 target splenocytes were incubated with SIINFEKL peptide (1 μg ml^−1^), OVA_323–339_ peptide (5 μg ml^−1^), or left unpulsed. Target cells were labelled with 25 μM CFSE (CFSE^HI^), 2.5 μM CFSE (CFSE^LO^) or 10 μM VPD450 and mixed together for injection into recipient mice (1/3 each SIIN-pulsed, OVA_323–339_ pulsed and unpulsed). Legend: each symbol represents a single mouse; (*n*=3–7 per group). Statistically significant differences were calculated by negative binomial regression. *P<0.05; **P<0.01; ***P<0.001; ****P<0.0001. Data are from two independent experiments. Error bars=s.e.m.

At this point the *in vitro* data indicated that the GMDC+B-cell combination might yield a superior anti-tumour response, based on slight increases in proliferation, 10-day fold expansion (data not shown), and IFN-γ production. We therefore compared the ability of lysate-loaded GMDC+B cell to stimulate *in vivo* cytotoxicity compared to GMDC. Effector T cells were generated as usual: cells were sorted pre-priming and isolated CD4^+^ or CD8^+^ T cells were cultured with lysate-loaded APCs. Target splenocytes pulsed with SIINFEKL or OVA_232–339_ were injected into WT mice. The following day CD4^+^ and CD8^+^ effector T cells primed with either soluble or oxidised lysate-loaded GMDCs or GMDCs+B cells were mixed 50/50 and adoptively transferred i.v. into mice that had received the targets. Twenty-four hours post-transfer spleens were collected and killing of peptide-loaded targets assessed by Flow Cytometry. Transferred CD4^+^ T cells elicited no killing (data not shown). Twenty-four hours after target transfer 45% of SIIN-pulsed cells were eliminated by GMDC+s-L-primed CD8^+^ T cells; 58% by GMDC+Bc+s-L-primed cells and 81% by GMDC+ox-L-primed cells (Figure 3). The greatest lysis (92%) occurred in the GMDC+Bc+ox-L-primed group (*P*<0.001). Thus, while oxidised lysate yielded weak enhancements of the CD8^+^ T-cell response *in vitro* when presented by the GMDC+B-cell combination, *in vivo* cytotoxicity demonstrated that CD8^+^ T cells primed with oxidised lysate conducted significantly more specific killing than those primed with soluble lysate. This enhanced killing occurred when oxidized lysate was presented by either GMDC alone, or by the GMDC+B cell combination. The GMDC+B cell combination elicited superior CD8^+^ T-cell-mediated killing when either soluble or oxidised lysate antigens were used as the antigen source, however, specific lysis was significantly higher in the oxidized lysate group (92%) than in the soluble lysate group (58% *P*<0.001).

### The combination of GMDC+B cell induces increased IL-12 production over GMDC alone

Having observed the increased proliferation, IFN-γ and *in vivo* cytotoxicity we assessed the IL-12 levels in combined APC cultures to ascertain a possible mechanism for the improved T-cell response. IL-12 secreted by activated DCs after CD40 ligation provides crucial cytokine information that helps drive T_H_1 differentiation and CTL effector function. We were therefore interested in the production of IL-12 by lysate-loaded APCs as an indicator of their capacity to stimulate IFN-γ production and promote T_H_1 skewing of T cells in response to lysate antigens.

**Figure 4:**
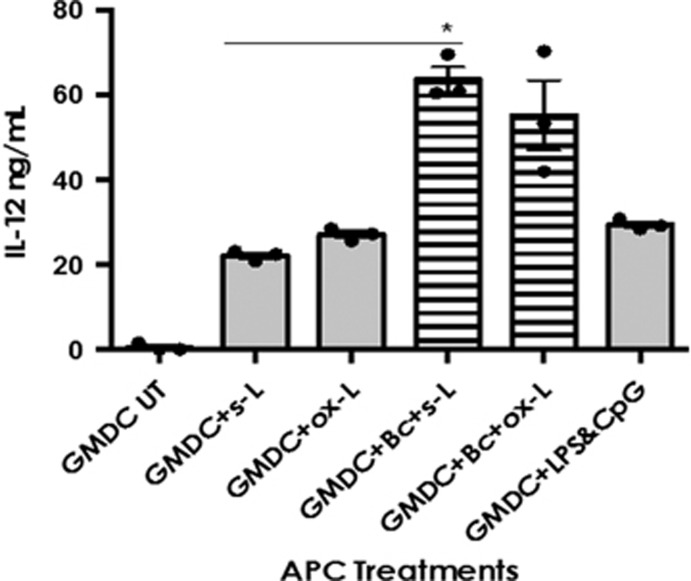
The combination of GMDC+B cell induces a synergistic increase in IL-12 production over both soluble and oxidised lysate-loaded GMDCs Day 6 GMDC, ±spleen-derived B cells, were pulsed overnight with whole OVA protein (50 μg ml^−1^) and s-L or ox-L (1:1 ratio, tumour cell:APC)+LPS&CpG. After 24 and 48 h cell conditioned media (supernatants) were collected and stored at −20 °C. Supernatants were analysed by anti-IL-12 ELISA. Summary data of three independent experiments showing IL-12 production after 48 h exposure to s-L and ox-L+LPS&CpG. Statistically significant differences calculated using Kruskal–Wallis followed by Dunn’s test for multiple comparisons with Bonferroni correction. *P<0.05. Error bars=mean±s.e.m.

GMDC produced significant amounts of IL-12 in response to both soluble and oxidised lysates (21 and 27 ng ml^−1^, respectively; Figure 4). However, as with MHC-II and CD40 upregulation, this IL-12 production was driven by the presence of LPS&CpG, rather than lysate material.

No improvement in IL-12 response was observed in the DC+Mϕ, Bc+Mϕ or Triple APC groups (data not shown). By contrast the combination of GMDC+B cell approximately tripled the production of IL-12 in soluble lysate-treated samples (64 ng ml^−1^; *P*<0.05). In oxidised lysate-treated samples IL-12 levels approximately doubled in the GMD+B cell group (55 ng ml^−1^; *P*=0.09). Importantly, IL-12 production in the GMDC+B-cell groups was greater than that observed in the GMDC+LPS&CpG groups, demonstrating IL-12 production beyond that which was achieved by LPS&CpG alone (28 ng ml^−1^). Taken together with the APC phenotype response, these IL-12 data suggested that the GMDC+B cell combination possessed an improved capacity for driving a desirable T-cell response to lysate antigens.

## Discussion

The optimal APC(s) for T-cell expansion in the context of adoptive cell therapy for cancer remains to be defined. The lack of robust results from clinical trials of monocyte-derived DCs is forcing researchers to examine alternatives such as peripheral blood-derived DCs from patients,^42^ and activated B cells.^29, 33, 34^ We wished to examine whether or not combining APCs, or stressing tumour cells prior to lysis, could enhance the T-cell response to tumour lysate antigens.

With regards to our aim of achieving a more immunostimulatory lysate, *in vitro* experiments showed differential costimulatory molecule responses to oxidised B16.OVA lysate according to which APC was exposed to the lysate: GMDCs upregulated CD40 on exposure to ox-L (*P*<0.05) but not s-L; but they did not upregulate MHC-II, CD80 or CD86. B cells meanwhile upregulated CD86 (*P*<0.05) in response to ox-L, but not MHC-II, CD40 or CD80. Taken together, these results suggest a capacity for enhanced T-cell responses to oxidized tumour lysate if both DCs and B cells are presenting the lysate antigens, as opposed to DCs alone, as is currently the case in the clinic.

B cell viability was improved in the presence of ox-L compared to s-L and subsequent data showed that T-cell viability was also improved when T cells were primed with APC loaded with ox-L rather than s-L (data not shown). Thus, while the ox-L had a negative impact on DC viability it had positive outcomes for B-cell and T-cell viability and we continued to evaluate its usefulness.

Mϕs downregulated MHC-II, and did not upregulate CD40, in response to either lysate. Thus, overall, the only improvement in immunogenic potential with oxidised lysate compared to soluble lysate was observed in the GMDC and B-cell groups. In a similar manner, in CD8^+^ T-cell proliferation assays, no statistically significant increase in proliferation over s-L was observed when ox-L was presented by GMDC, although greater proliferation did occur when oxidised lysate was presented by GMDC+B cells.

In the *in vivo* cytotoxicity assays, however, priming T cells with oxidised lysate proved significantly more immunogenic than soluble lysate whether presented by GMDC or GMDC+B cell. Thus in these *in vivo* experiments oxidation provided an effective means of increasing the immunogenicity of poorly immunogenic molecules, irrespective of the APC(s) presenting the lysate antigens. Nonetheless, the greatest cytotoxicity was observed in the DC+B-cell groups.

DC-B-cell cooperation and B-cell capacity to act as APCs has been observed by other investigators. Shirota *et al.* challenged the notion that B cells lack antigen non-specific capture, priming of naïve T cells and T_H_1 induction capacity.^43^ Their study demonstrated that in the presence of OVA antigen conjugated to CpG, B cells assumed the same functional capacity as DCs. In this current study the use of unconjugated CpG, along with LPS, during APC loading with soluble and oxidised lysates, resulted in increased MHC-II and CD40 (DCs), and IL-12 production (all conditions) when compared to lysates alone. These data indicated, respectively, increased antigen presentation capacity, co-stimulatory capacity and the ability to drive T_H_1 IFN-γ responses. Interestingly, however, while the B cell MHC-II response was enhanced by the presence of LPS&CpG, B cell CD86 was upregulated in the absence of LPS&CpG, therefore this upregulation was driven by unidentified factors in the lysate. We assume that stress molecules upregulated or released by the oxidised tumor cells stimulated this response in the B cells, however, the molecules that triggered this response remain to be identified. Nevertheless these data provide further insight into the exquisitely specific nature of the immune response to various immune stimuli. The DCs and B cells did not respond in an identical manner to the same stimuli. If we understand how each APC responds to a given stimuli, we can harness this knowledge for rational design of inter-cellular cooperative immune activation strategies.

Another intriguing example of CpG-mediated anti-tumour effect was reported after intratumoural injection of the TLR9 agonist CpG, along with anti-OX40L and/or anti-CTLA4 antibodies, to eliminate T_REG_s in the tumour microenvironment. This approach initiated a systemic anti-tumour response and long-lasting protection in mice.^44^ Conversely, systemic delivery of antibodies and CpG ODNs had an immediate anti-tumour effect but mice later relapsed. The study authors attributed the results to the depletion of T_REG_s by mABs but in light of our results, it seems plausible that the anti-tumour effect observed in the intratumoural injection groups might have arisen from the effects of damaged tumour cells being exposed to activated DCs and B cells in the tumour, which were cooperatively able to prime the CTLs more effectively.

In summary, we have shown that oxidised lysate-loaded GMDC alone demonstrated superior presentation and costimulatory capacity over Mϕs and B cells. We have also shown that when the GMDC population was combined with B cells the potential for driving a T_H_1 T-cell response was markedly improved via an increase in IL-12 production. The GMDC+B cell combination was not superior to GMDC alone at stimulating CD8^+^ T cell proliferation when presenting soluble lysate. However, greater CD8^+^ T-cell proliferation was achieved when the T cells were primed with oxidised lysate-loaded GMDC+B cells.

These data have demonstrated an improved CD8^+^ T-cell response to priming by GMDC+B cell and opens up the potential for translation into the clinic. Currently, GM-CSF-differentiated monocyte-derived DCs are the APC of choice for *ex vivo* priming and expansion of T cells against defined or undefined tumour antigens in the clinic.^45^ However, the inconsistent results with mo-DCs is necessitating re-examination of the use of patient blood DCs,^42^ despite their comprising <1% of human PBMCs.^46^ B cells possess many of the same biological attributes as DC, including high MHC expression and antigen-specific T-cell-regulating cytokine profiles. These reasons, along with their relative ease of use, are bringing them to the attention of investigators as alternatives to DCs in immunotherapy.^34^ Autologous B cells vastly outnumber DCs and can be readily expanded to even greater numbers from patient blood.^33^ Monocyte-differentiated DCs are significantly lower in number than B cells to begin with, and cannot be expanded as required for multiple transfers in therapeutic schedules.^31, 47, 48^ Reinfused B cells migrate to lymph tissue, the principal site of T-cell activation and, depending on their antigenic load, can avoid CTL-mediated destruction that can plague antigen delivery by infused DCs. In the LNs B cells can deliver antigen to follicular DCs which in turn present antigen to CD8^+^ CTLs and T_CM_ cells. Indeed, novel ways to enhance antigen-non-specific uptake by B cells are already being explored.^49^ Our group will shortly begin experiments to explore CD40L-activated B cells and GM-CSF-differentiated DCs in human diffuse intrinsic pontine glioma (DIPG). We wish to ascertain if the enhanced T-cell response observed in this current study can be replicated in humans in a different cancer type, or whether it was unique to this murine melanoma model. Just as combined immunotherapies like the checkpoint blockade inhibitors have proved superior in cancer treatment, the use of combined APCs may also enter the clinic in the near future.

## Methods

### Animals and cell lines

Six to 16-week-old male and female C57BL/6 mice were obtained from the Hercus Taieri Resource Unit, Mosgiel, Dunedin, Otago, New Zealand. Mice were maintained in specific pathogen-free conditions, and studies were performed in accordance with local ethical guidelines. The B16-OVA cell line was maintained in culture in RPMI1640 (Gibco, Thermo Fisher Scientific, Waltham, MA, USA) supplemented with 10% heat-inactivated FCS (Moregate Biotech, Hamilton, New Zealand), 100 U ml^−l^ penicillin and 100 μg ml^−l^ streptomycin (Gibco, Thermo Fisher Scientific) and 1% Geneticin (Gibco, Thermo Fisher Scientific). The line was tested and found to be mycoplasma-free.

### Oxidation of cell lines

To induce and detect oxidation-dependent necrotic tumour cell death the protocol developed by Chiang *et al.*^15^ was followed. Briefly, B16.OVA cells were treated for 5 h with BrefeldinA to allow intracellular OVA accumulation. To collect the cells the medium was discarded, the flasks rinsed with 0.02% EDTA solution and the adherent cells incubated with 0.02% EDTA solution for ~5 min. Cells were rinsed from the flasks, washed and resuspended in DPBS. Stock NaOCl reagent (Sodium Hypochlorite 12.0–15% Solution, ecplabchem.co.nz) was added to the tumour cells to give a final cell density of 8 × 10^5^ cells per ml in a 90 μM HOCl solution (90 μM had previously been determined to generate 95% cell death). Tumour cell suspensions were incubated for 1 h at 37 °C/5% CO_2_ with gentle agitation at 30 min to induce oxidation-dependent tumour cell death. Following HOCl treatment, tumour cells were washed twice with DPBS prior to undergoing 6 rounds of freeze–thaw lysis (methanol dry-ice bath 20 min; 37° water bath 5 min). Cell death was verified by Trypan Blue exclusion and Propidium iodide (PI) staining was used to quantify the percentage of dead cells by Flow Cytometry.

### Generation of bone marrow-derived GMDC and bone marrow-derived macrophages

Bone marrow cells were isolated from femurs and tibiae of C57/BL6 mice according to well-established protocols. Briefly, the intact bones were sterilised in 70% ethanol (2 min), the epiphyses removed and the shafts flushed with DPBS to extract the marrow. Red blood cells were lysed with ACK RBC lysis buffer (2 ml, 3 min) and cells resuspended in medium for counting and plating. To generate GMDCs precursor cells were resuspended in IMDM+5% FCS+20 ng ml^−1^ GM-CSF and 2.5 × 10^6^ cells plated in 4 ml medium in six-well plates. Differentiating GMDCs were fed on D3 by removing 2 ml of medium and replacing with 2 ml warm, fresh medium. To generate Mϕs precursor cells were resuspended at 1 × 10^6^ c per ml in IMDM+10% FCS+5 ng ml^−1^ GM-CSF and plated overnight in T75 or T175 tissue culture flasks to allow fibroblast adherence. The following day 5 ng ml^−1^ IL-3 was added and the non-adherent cells transferred to 100 mm sterile bacteriological petri dishes (5 ml per dish). On Days 4 and 7 the differentiating cells were gently rinsed with the medium in the dish. The non-adherent cell suspension was discarded and replaced with fresh, warm 5 ml of medium. Day 6 GMDC and Day 10 Mϕs were pulsed with lysates or whole OVA protein and LPS&CpG overnight prior to the addition of T cells.

### Isolation of B cells

The spleens of 6–12-week-old C57BL/6 mice were isolated asceptically and placed in Dulbecco’s phosphate-buffered saline (DPBS; Gibco, Paisley, Scotland). In a laminar flow hood the plunger from a sterile syringe was used to gently press the spleen through a 70 μm cell filter sitting in a petri dish of DPBS. The disaggregated cell suspension was centrifuged (7 min, 300 *g*, 4 °C). The supernatant was discarded, and the cells resuspended in ACK RBC lysis buffer (2 ml, 3 min) to lyse red blood cells. Residual cells were washed in DPBS and prepared for magnetic cell isolation with anti-CD43 (Ly-48) MicroBeads (Miltenyi Biotec GmbH, Bergisch Gladbach, Germany) as per the manufacturer’s instructions. Cells were passed through a large cell column on an AutoMACS Pro Separator (Miltenyi Biotec GmbH, Germany) ‘DepleteS’ programme. The negative fraction was, resuspended in B cell medium (Iscove’s Modified Dulbecco’s Medium plus 10% FBS; IMDM10) and counted. The positive and negative fractions were stained for B220 (CD45R), CD19, CD3 and CD11c to check for purity and DC or T-cell contamination. Spleen-derived B cells were pulsed with whole protein and LPS&CpG overnight prior to the addition of T cells.

### Phenotypic analysis of DCs, Mϕs and B cells exposed to HOCl-oxidized tumour cells

To investigate the effects of HOCl-oxidized tumour cells on APC maturation, tumour cells were treated as described previously and co-cultured with APCs at a 1:1 or 2:1 ratio (2 APCs to 1 tumour cell) for 24 h at 37 ° C+5%CO_2_. After incubation, APCs were collected and stained with the viability dye Live/Dead Near Infra-Red (Life Technologies, Thermo Fisher Scientific, MA, USA), FITC-conjugated anti-IA/IE (MHC-II) (Clone: M5/114.15.2, BioLegend), PE-conjugated CD135 (Clone: A2F10, BioLegend, San Diego, CA, USA), CD115 (Clone: AF598, BioLegend), CD169 (Clone: 3D6.112, BioLegend), F4/80 (Clone: BM8, BioLegend), CD19 (Clone 6D5, BioLegend), CD45R (B220)-PerCP-Cy5.5 (Clone RA3-6B2, BioLegend), CD8-APC-H7 (Clone: 53-6.7, BioLegend), CD11c-APC (Clone: N418, BioLegend), BV421-CD206 (C068C2, BioLegend) and the mAbs for APC maturation markers CD80-BV421(Clone: I6-10A1, BioLegend) and CD40-APC (Clone: 3/23, BioLegend). Briefly, media were removed and Mϕs rinsed in 0.02% EDTA solution then incubated in 0.02% EDTA solution for 5 min at room temperature to release Mϕs from non-tissue culture-treated plastic ware. APCs were washed and resuspended in room temperature DPBS for viability staining (15 min, room temperature). Fc Receptor block was also added at this time. Cells were washed and resuspended in cold FACS staining buffer (1 × PBS, 0.1% bovine serum albumin, 0.01% sodium azide (NaN_3_)) and the relevant mABs were added for 15 min at 4 °C, followed by two washes with cold FACS staining buffer. Cells were fixed in 4% paraformaldehyde, acquired the following day on a Gallios flow cytometer (Beckman Coulter, Brea, CA, USA) and the data analyzed using FlowJo software (Version 10, TreeStar, Ashland, OR, USA).

### Cytokine production

Lysate-loaded APCs were cultured for 24–72 h. IL-12 levels were assayed by anti-IL-12 ELISA using Nunc-Immuno Maxisorp 96 well plates (Thermo Fisher Scientific, Roskilde, Denmark), purified anti-mouse IL-12, recombinant mouse IL-12 and biotinylated anti-mouse IL-12 (all from BioLegend).

### T-cell isolation

The spleens of 6–12-week-old OT-I and OT-II mice were isolated asceptically and placed in sterile DPBS (Gibco, Paisley, Scotland). In a laminar flow hood, on an ice tray, spleens were gently pressed through a 70 μM cell filter using the plunger from a sterile syringe. Cell aggregates were dissociated by flushing the cell suspension through the sieve with a 10 ml pipette. The cell suspension was centrifuged (7 min, 300 × g, 4 °C), and red blood cells lysed by resuspending the cell pellet by gentle pipetting in ACK RBC lysis buffer (5 ml, 3 min). Residual cells were washed in DPBS and resuspended in 40 μl of AutoMACS buffer per 10^7^ cells. Antibody cocktails (anti-CD4 and anti-CD8-negative selection kits; Miltenyi Biotec GmbH) were vortexed and 10 μl of antibodies added per 10^7^ cells. The antibody-labelled cell suspension was briefly vortexed and incubated at 4 °C for 5 min. AutoMACS buffer was added (30 μl per 10^7^ cells), and anti-biotin beads (20 μl per 10^7^ cells). The bead/cell suspension was incubated at 4 °C for 10 min, and topped up with AutoMACS buffer to 500 μl per 10^8^ cells (if necessary). Cells were passed through a large cell column on an AutoMACS Pro Separator (Miltenyi Biotec GmbH) ‘DepleteS’ programme. The negative fraction was collected, resuspended in DPBS and counted. The negative fractions were stained for the surface molecules CD3, CD8 or CD4, CD28, CD27, PD-1, CD44, CD122, CD127, CD62L, CD157 (CTLA-4) and CCR7 to assess the naive and T-cell status.

### Proliferation assay

Working in a hood with the light off T cells were resuspended at 5 × 10^7^ cells per ml. A 10 μM (2 ×) CFSE solution was prepared by adding 1 μl of 10 mM stock to 1 ml DPBS. This solution was added to an equal volume of cell suspension for a final concentration of 5 μM CFSE. The tube was immediately vortexed briefly, wrapped in foil and incubated for 5 min at room temperature on a ground rotator to ensure even staining. Labelled cells were washed twice by diluting in 10 volumes of room temperature DPBS+5% FBS followed by sedimentation by centrifugation (300 *g*, 5 min, 20 °C). Labelled cells were resuspended in T-cell medium (Advanced DMEM+5% FBS+HEPES (1%)+GlutaMax (1%) +/− IL-2 (5 ng ml^−1^) ±α-CD28 (2 μg ml^−1^) +/− IL-7, IL-15, IL-21 (all 100 μg ml^−1^) at 10–20 × 10^6^ cells per ml for plating with APCs (ratio 10T cells:1 APC). After 72 h of co-culture cells were collected, stained with live/dead exclusion dye and with surface mABs prior to fixation in 4% paraformaldehyde. Fixed cells were stored overnight at 4 °C and acquired the following day on a Gallios Flow Cytometer (Beckman Coulter). Data were analysed on FlowJo VX (TreeStar). Doublets were first gated out off the forward scatter height (FSC-H) versus side scatter area (SSC-A) plot. Dump channels were used to gate out dead cells (viability dye positive cells) as well as any APCs (CD11c and CD19 in DC and B cell co-cultures, respectively) that may have been harvested along with the T cells. Due to the highly adherent nature of Mϕs gating out CD11c+ cells was unnecessary in Mϕ-T-cell co-cultures. Finally, the cells in the CD3^+^ CD8^+^, or CD3^+^ CD4^+^ double-positive quadrants were selected and analysed for CFSE+ proliferation. Graphs and statistical analyses were performed in Graph Pad Prism (GraphPad Software Inc, San Diego, CA, USA).

### *In vivo* cytotoxicity

These experiments were approved by the Animal Ethics Committee, University of Otago and conducted under Animal Ethics approval number AEC59/15. APCs and T cells were generated as described above. GMDCs±B cells were loaded with lysate and activated with LPS&CpG. The following day CD4^+^ OT-II and CD8^+^ OT-I T cells were isolated and added to the lysate-loaded APCs at a ratio of 10T cells to 1 APC. At Day 3 or 4, effector T cells were collected, washed twice in sterile DPBS, counted and resuspended at 1 × 10^7^ cells per ml. Effector cells were adoptively transferred into C57BL/6 female mice via i.v. injection into the tail vein (200 μl per mouse). The following morning target cells were prepared. Splenocytes isolated from C57BL/6 mice were divided into three tubes, pulsed with 1 μg ml^−l^ OVA_257–264_ peptide or 5 μg ml^−l^ OVA_323–339_ (SIINFEKL) peptide, or left unpulsed as a control against non-specific killing. Peptide-loaded cells were incubated at 37 °C for 3 h, washed three times in DPBS and the concentrations adjusted to 5 × 10^7^ cells per ml for CFSE and VPD450 labelling. SIINFEKL-pulsed cells were stained with 25 μM CFSE (CFSE^HI^) and OVA_257–264_ peptide-pulsed cells were stained with 2.5 μM CFSE (CFSE^LO^) to allow identification of which targets were killed. Unpulsed cells were stained with 10 μM VPD450. CFSE and VPD450 labelling was carried out as described above.

Dye-stained cell concentrations were adjusted to 50 × 10^6^ cells per ml in DPBS. Equal volumes of the 50 × 10^6^ c per ml target cell types (unpulsed, SIIN-pulsed & OVA_323–339_-pulsed) were mixed together and 200 μl (1 × 10^7^ target cells total; 3.33 × 10^6^ per target type per mouse) was drawn into sterile syringes and injected into the tail vein of recipient mice. Mice were killed 17–24 h after adoptive transfer of target cells. Spleens were isolated and splenocyte single cell suspensions prepared. Splenocytes were labelled with dead cell exclusion dye (Live/Dead Near InfraRed; Life Technologies), fixed with 4% paraformaldehyde, stored overnight at 4 °C and acquired the following day on a Gallios Flow Cytometer (Beckman Coulter). The percentage lysis of target cells was calculated using the formula:



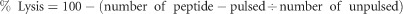



### Statistical analysis

Analyses were performed and graphs created in Graph Pad Prism (GraphPad Software Inc, San Diego, CA, USA). A significance level of 0.05 or less was considered statistically significant. The Kruskal–Wallis non-parametric comparison of location was applied for comparisons of three or more groups. Dunn’s test for multiple comparisons, with Bonferroni correction, was applied *post hoc* to the rank sums to calculate differences between the groups. This conservative analysis makes any statistically significant result very robust.

Differences between treatments for *in vivo* cytotoxicity assays were analysed using negative binomial regression, which accounts for an overdispersed Poisson distribution. The model included terms for the groups and adjusted for the number of cells collected. No adjustment was made for multiple comparisons, however, all *P* values were <0.0001 indicating that the results are highly unlikely to be due to chance.
