# Supplementary material for: Combining dendritic cells and B cells for presentation of oxidised tumour antigens to CD8+ T cells
Source: Clin Transl Immunology. 2017 Jul 7;6(7):e149–. doi: 10.1038/cti.2017.28 (PMC5539416; doi:10.1038/cti.2017.28)
Supplement: Supplementary Figures [file cti201728x1.pdf]

Supplementary Figure 1

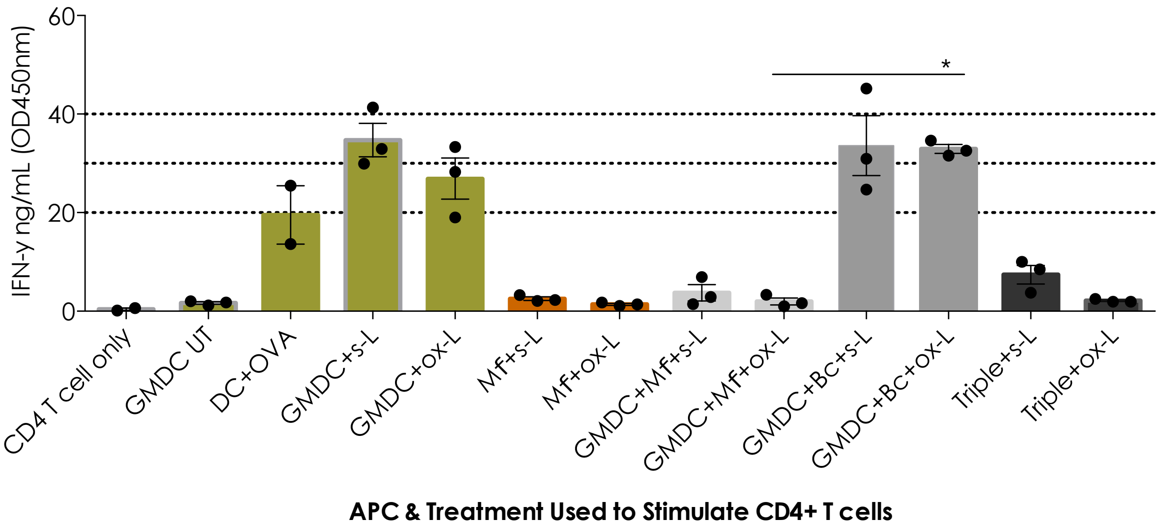

Supplementary Figure 1. GMDC+B cell induces no increase in CD4+ T cell IFN-γ production over GMDC alone when presenting lysate antigens. Day 6 GMDC, Day 10 MΦ and freshly isolated splenic B cells (CD43- cells), or combinations thereof, were pulsed overnight with whole OVA protein (50 μg/mL) and B16.OVA s-L or ox-L (1:1 ratio, tumour cell:APC). LPS (1 μg/mL) and CpG (0.3 μg/mL) were added at the same time as the lysates. The following morning CFSE-labelled CD4+ T cells were added (1:10 ratio, APC:T cell). APCs and T cells were co-cultured for 72 hours, conditioned cell media collected prior to cell harvest and stored at -20°C. IFN-γ levels were assessed by anti-IFN-γ ELISA. Data was analysed in Exel and graphed in Prism (GraphPad, San Diego, CA, USA). Summary data of 3 independent experiments plated in duplicate. Statistically significant differences calculated using Kruskal-Wallis followed by Dunn's test for multiple comparisons with no Bonferroni correction * $p<0.05$. Error bars = mean ± s.e.m.

Supplementary Figure 2

A

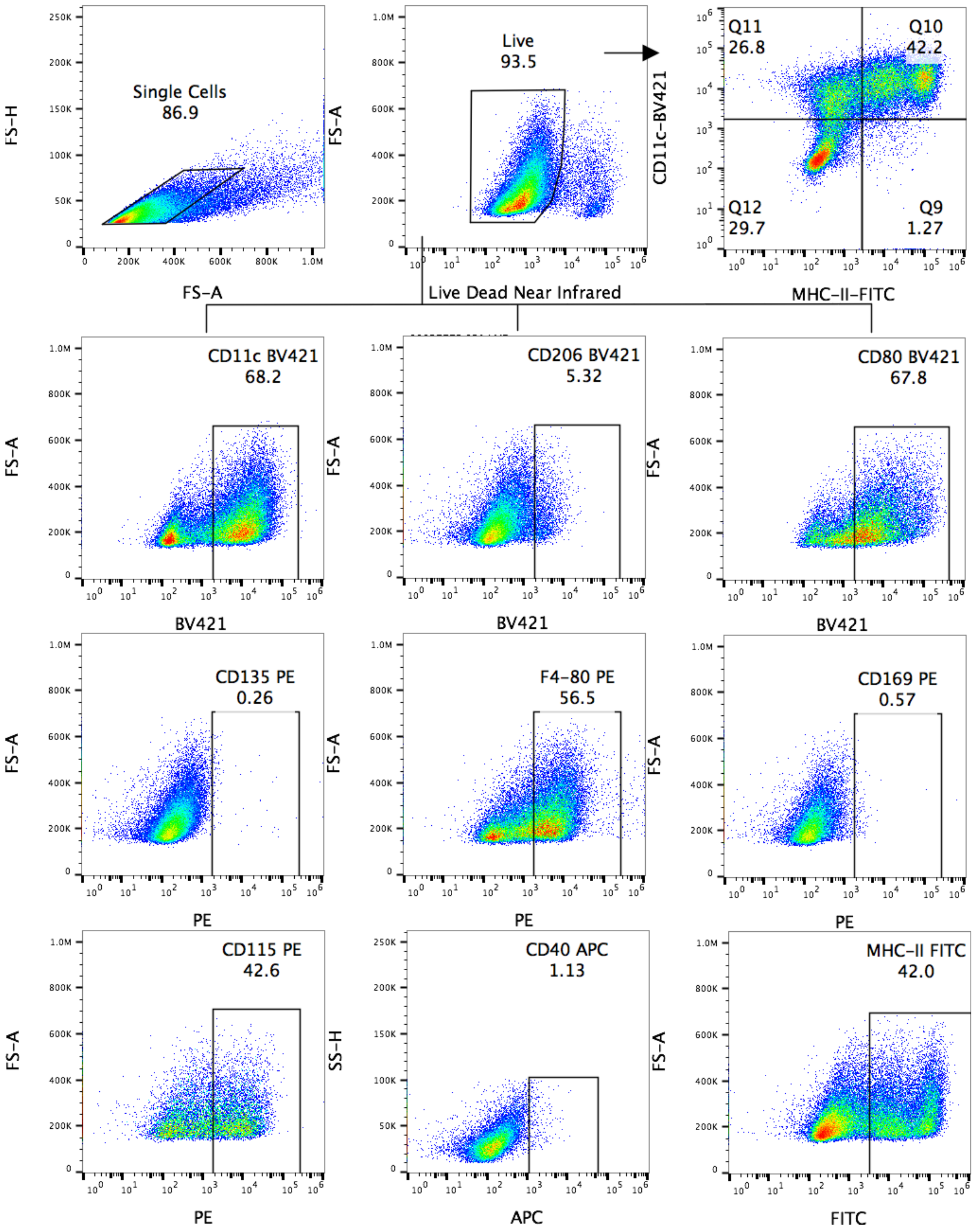

B

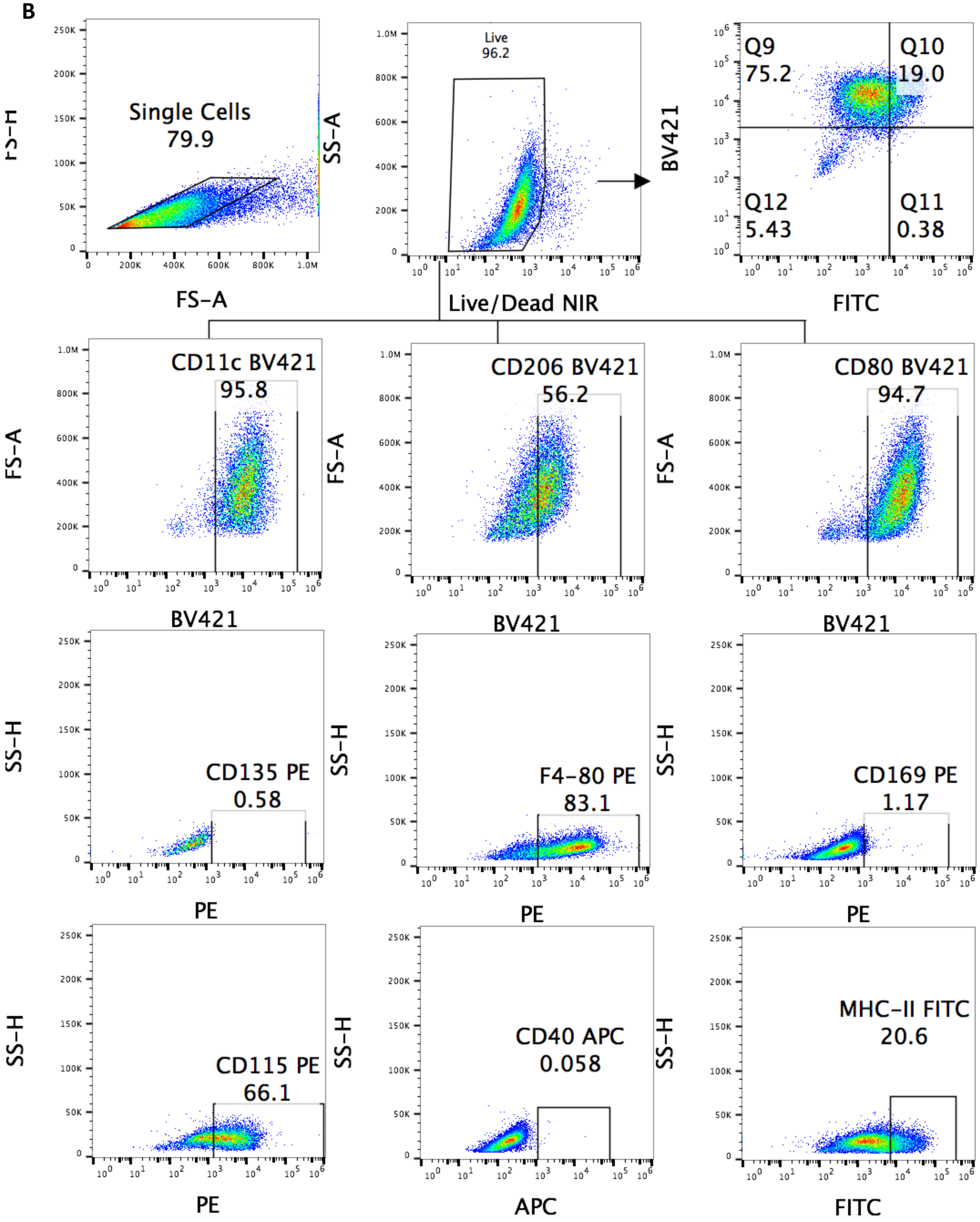

Single Cells
79.9
FS-A
Live
96.2
Live/Dead NIR
BV421
Q9
75.2
Q10
19.0
Q12
5.43
Q11
0.38
FITC
CD11c BV421
95.8
CD206 BV421
56.2
CD80 BV421
94.7
FS-A
BV421
CD135 PE
0.58
F4-80 PE
83.1
CD169 PE
1.17
SS-H
PE
CD115 PE
66.1
CD40 APC
0.058
MHC-II FITC
20.6
APC
FITC

Supplementary Figure 2. Gating Strategy Used in DC and MΦ Phenotype Analysis. D6 GMDCs and D10 M1 MΦ were prepared as previously described. Cells were stained with Live/Dead exclusion dye and Fc receptor block followed by surface molecule staining with the following monoclonal antibodies: MHC-II-FITC, CD11c-BV421, CD135-PE, CD169-PE, CD115-PE, F4/80-PE, CD40-APC, CD206-BV421, CD80-BV421. Antibody-stained cells were washed twice and fixed in 4% paraformaldehyde. Cells were stored overnight at 4°C and acquired the following day on a Gallios Flow Cytometer. 5×10^4 - 1×10^5 cells were collected per sample wherever possible. The gating strategy consisted of doublet exclusion followed by dead cell exclusion and then gating on the cells of interest using unstained cells and single stained cells as negative gating controls. Data was analysed and graphed in FlowJo software Version X. Representative data from numerous independent experiments. A) GMDC; B) MΦs

Supplementary Figure 3

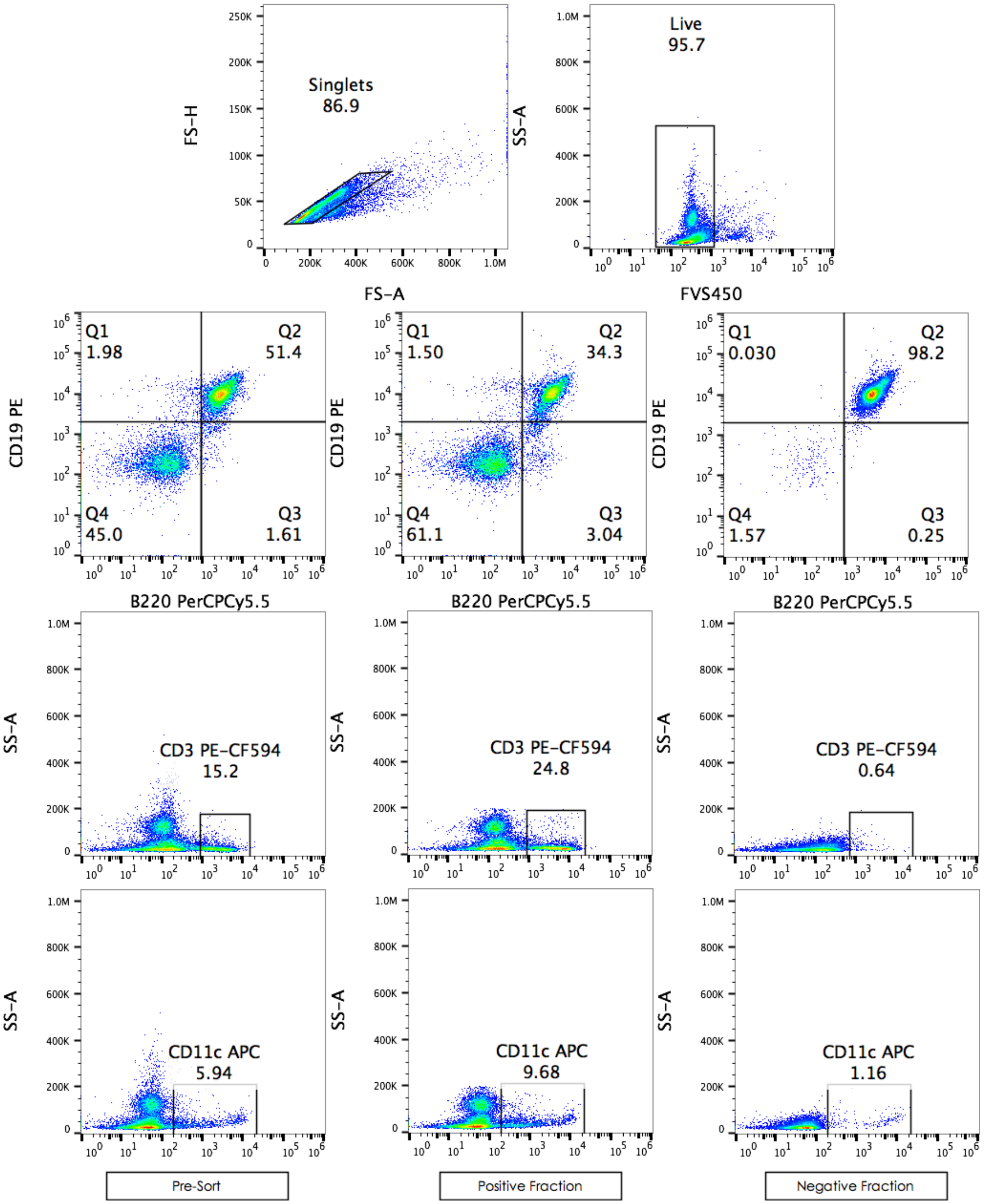

Supplementary Figure 3. C57BL/6 splenocytes labeled with anti-CD43 magnetic beads yield up to 98% pure B cell populations. Splenocytes from WT C57BL/6 mice were lysed with RBC lysis buffer and incubated with anti-CD43 magnetic beads as per the manufacturer's instructions (Miltenyi Biotech). Cells were subjected to a negative selection

protocol on an AutoMACS Pro and the resulting populations stained with Lived/dead exclusion Dye (FVS450; BD Biosciences). Cells were incubated with Fc receptor block prior to incubation with monoclonal antibodies against CD11c (APC), CD3 (PE-CF594), CD19 (PE) and B220 (PerCPCy5.5) (all from BioLegend). Cells were fixed with 4% PFA and stored overnight at 4°C. Cells were processed the following day on a Gallios Flow Cytometer (Beckman Coulter) and the data analysed on FlowJo Version 10 or Flow Jo 2 (TreeStar, Ashland, OR, USA). Representative Flow Cytometric dot plots from multiple independent experiments
